# Optimization of Engine Piston Performance Based on Multi-Method Coupling: Sensitivity Analysis, Response Surface Model, and Application of Genetic Algorithm

**DOI:** 10.3390/ma18133043

**Published:** 2025-06-26

**Authors:** Bin Zheng, Qintao Shui, Zhecheng Luo, Peihao Hu, Yunjin Yang, Jilin Lei, Guofu Yin

**Affiliations:** 1 School of Intelligent Manufacturing, Panzhihua University, Panzhihua 617000, China; lucky20000604@163.com (Q.S.); 17390366120@173.com (Z.L.); 15708329745@163.com (P.H.); yyj021122@163.com (Y.Y.); 2 Yunnan Province Key Laboratory of Internal Combustion Engines, Kunming University of Science and Technology, Kunming 650500, China; leijilin@kmust.edu.cn; 3 School of Manufacturing Science and Engineering, Sichuan University, Chengdu 610065, China; gfyin@scu.edu.cn

**Keywords:** piston, thermal–mechanical coupling analysis, modal analysis, harmonic response analysis, response surface analysis, ANOVA, optimization design

## Abstract

This paper focuses on the use of advanced optimization design strategies to improve the performance and service life of engine pistons, with emphasis on enhancing their stiffness, strength, and dynamic characteristics. As a core component of the engine, the structural design and optimization of the piston are of great significance to its efficiency and reliability. First, a three-dimensional (3D) model of the piston was constructed and imported into ANSYS Workbench for finite element modeling and high-quality meshing. Based on the empirical formula, the actual working environment temperature and heat transfer coefficient of the piston were accurately determined and used as boundary conditions for thermomechanical coupling analysis to accurately simulate the thermal and deformation state under complex working conditions. Dynamic characteristic analysis was used to obtain the displacement–frequency curve, providing key data support for predicting resonance behavior, evaluating structural strength, and optimizing the design. In the optimization stage, five geometric dimensions are selected as design variables. The deformation, mass, temperature, and the first to third natural frequencies are considered as optimization goals. The response surface model is constructed by means of the design of the experiments method, and the fitted model is evaluated in detail. The results show that the models are all significant. The adequacy of the model fitting is verified by the “Residuals vs. Run” plot, and potential data problems are identified. The “Predicted vs. Actual” plot is used to evaluate the fitting accuracy and prediction ability of the model for the experimental data, avoiding over-fitting or under-fitting problems, and guiding the optimization direction. Subsequently, the sensitivity analysis was carried out to reveal the variables that have a significant impact on the objective function, and in-depth analysis was conducted in combination with the response surface. The multi-objective genetic algorithm (MOGA), screening, and response surface methodology (RSM) were, respectively, used to comprehensively optimize the objective function. Through experiments and analysis, the optimal solution of the MOGA algorithm was selected for implementation. After optimization, the piston mass and deformation remained relatively stable, and the working temperature dropped from 312.75 °C to 308.07 °C, which is conducive to extending the component life and improving the thermal efficiency. The first to third natural frequencies increased from 1651.60 Hz to 1671.80 Hz, 1656.70 Hz to 1665.70 Hz, and 1752.90 Hz to 1776.50 Hz, respectively, significantly enhancing the dynamic stability and vibration resistance. This study integrates sensitivity analysis, response surface models, and genetic algorithms to solve multi-objective optimization problems, successfully improving piston performance.

## 1. Introduction

Internal combustion engines, as one of the most important power sources today, are widely used in automobiles, ships, aircraft, and other fields. However, with the increasingly severe global energy crisis and environmental pollution problems, the requirements for the performance of internal combustion engines are also constantly increasing. Pistons, as one of the most critical components in internal combustion engines, work under extremely harsh conditions, not only having to withstand the impact of high-temperature and high-pressure gas but also experiencing rapid reciprocating motion and heat exchange processes. These complex working conditions place extremely high demands on the material, structure, and cooling system of the piston. Hence, through thermal–mechanical coupling analysis, dynamic characteristics research, and structural optimization, the power performance and fuel economy of the engine are improved. In the meantime, the wear is effectively reduced, and the service life is prolonged.

With the rapid development of computer technology and numerical analysis methods, advanced technologies such as finite element analysis (FEA) and computational fluid dynamics have been widely used in piston design and optimization. Through FEA, the stress distribution, deformation, and temperature field characteristics of the piston under complex working conditions can be accurately simulated, providing a scientific basis for the structural optimization. By using modal analysis, the dynamic performance can be deeply understood, and potential resonance risks can be predicted, providing a basis for vibration avoidance design. However, the thermomechanical coupling analysis and optimization research of the piston still faces many challenges. The working process of the piston involves the coupling effect of multiple physical fields, such as the interaction of the temperature field, stress field, and fluid field, which makes the analysis process extremely complex. The optimization design needs to consider multiple objective functions and constraints, such as strength, stiffness, mass, cost, etc. Therefore, how to find the optimal solution among these conflicting objectives is a current research hotspot.

Nowadays, some progress has been made in the research on the performance optimization and failure mechanism of pistons under complex working conditions. Regarding the wear problem of the titanium alloy piston skirt, through the application of metallographic analysis, electron microscope scanning, and FEA (ABAQUS), it was revealed that the deformation caused by insufficient stiffness of the piston skirt was the main failure reason, and the wear failure problem was solved through structural parameter optimization [1]. The experimental results show that the research on the thermal insulation performance of the composite adiabatic piston has achieved remarkable results. The results show that the application of the thermal insulation pad and air gap has a significant effect on improving the thermal insulation effect of piston. It has been proven that the finite element model has high-precision prediction performance [2]. In the exploration of piston cooling and strength optimization, the design research of the internal cooling oil cavity reveals its complex influence on piston heat transfer and structural strength, and the orthogonal test design method effectively evaluates the optimization potential of factors such as oil cavity type and surface area [3]. For high-strength diesel engine pistons, through SolidWorks modeling and FEA, the thermal stress, thermal deformation, and stress concentration areas of the piston under high-temperature and high-pressure environments are discussed in detail, providing a scientific basis for structural improvement [4]. After thermal boundary condition analysis, temperature field simulation, and experimental verification, an innovative design scheme of aluminum skirt piston with steel top came into being. In this scheme, a composite heat-insulating piston with air gap and heat-insulating gasket was proposed to reduce the heat flow output in the piston ring groove area. Through structural optimization and adjustment, the contact stress distribution between the thermal insulation gasket and the piston top is greatly improved. These research results not only deepen the understanding of the performance and failure mechanism of diesel engine pistons but also point out the direction for future piston design and optimization [5].

The thermal fatigue life and wear resistance of pistons, as key components, have become a research hotspot. Regarding the influence of uneven temperature distribution on the top surface during the intake process on fatigue life, researchers have successfully predicted the fatigue life through CFD simulation, FEA, and material testing and revealed the significant impact of intake cooling effects on its fatigue life [6]. In addition, for the change in the thermal load of the piston with different working conditions, especially the characteristics of thermal stress and thermal strain under cold start and other working conditions, a detailed analysis has been carried out, providing a theoretical basis for the design of high-strength diesel engine pistons [7]. Furthermore, in order to improve the wear resistance of the piston skirt, a bionic through-hole structure based on the bionic non-smooth theory was designed, as well as through FEA and bench tests. It significantly reduced skirt wear and optimized the oil film retention ability [8].

The friction loss of the internal combustion engine piston–cylinder liner system, as a key factor affecting engine energy efficiency and emissions, has received widespread attention [9,10,11]. To effectively reduce this loss, researchers have drawn inspiration from nature and applied the shell structure to the design of the piston skirt. Through orthogonal experimental design, combined with durability bench tests, the significant effect of the bionic structure in improving the wear resistance of the piston, reducing the frictional resistance, and improving the stability of the cylinder pressure has been confirmed. This research shows that the application of a shell stripe structure can reduce the wear of the piston skirt. It also optimizes the stress distribution and prolongs fatigue life. The working temperature is significantly reduced.

With the continuous progress of engine technology, heat loss and emission issues have become the key factors restricting the efficiency and lifespan of engines. To address this challenge, thermal ceramic coatings are regarded as an important means of improving engine performance due to their excellent heat insulation and wear resistance. In several studies, zirconium carbide ceramic coatings have been applied to the pistons and cylinders of V12 engines, and FEA reveals that they significantly reduce the heat flux and effectively block heat transfer, thereby improving engine efficiency and extending the lifespan of parts [12]. With the increase in diesel engine power, pistons are faced with more severe thermal and mechanical loads, and their fatigue life has become the focus of design. With the help of advanced simulation tools such as ANSYS, researchers have deeply analyzed the thermomechanical coupling behavior of pistons under complex working conditions to ensure that the design meets the strength requirements, providing a theoretical basis for improving the overall performance of diesel engines [13]. Through topology optimization and metal additive manufacturing technology, the innovative design of the compressor piston has been realized, significantly reducing the amount of materials used and improving efficiency, demonstrating the great potential of advanced manufacturing technology in improving the performance of turbo-machinery [14]. In response to the mechanical failure problem of commercial vehicle diesel engines at high-temperature torque points, ceramic coating technology has once again demonstrated its application value. Studies on the effect of different thicknesses of ceramic coatings on the piston temperature distribution show that increasing the coating thickness can reduce temperature [15].

Research on Al-Si alloy piston materials shows that through the optimization of extrusion casting and heat treatment processes, the thermal conductivity and strength of the materials can be significantly improved, providing new ideas for the selection and processing of piston materials [16]. The research on aluminum–silicon (Al-Si) alloy piston materials has been continuously deepened, and especially significant progress has been made in the influence of alloying elements on thermal properties. By measuring the influence of the addition of different alloying elements (such as Cu and Ni) on key parameters such as the thermal diffusivity of Al-Si alloys, and combining experiments with finite element simulations, the important role of alloy composition on the surface temperature and overall thermal performance has been revealed. These studies indicate that reasonable control of the content of alloying elements can effectively adjust the heat resistance and high-temperature performance of piston materials, thereby improving the operating efficiency and reliability of the engine [17].

The piston shape design, as one of the key factors that improve the performance of diesel engines, has also received widespread attention. By comparing and analyzing the characteristics of different piston shapes—such as flat, domed, cup-shaped, and bowl-shaped—in terms of thermal stress, temperature distribution, and total deformation, it is found that flat and domed pistons perform better in reducing thermal stress and total deformation [18]. To further improve the reliability of diesel engine and prolong its service life, researchers have also optimized the piston geometry based on FEA. By comprehensively considering the influence of geometric parameters such as intake depth and exhaust valve groove on the temperature and stress, significant improvements in piston performance have been achieved using advanced optimization algorithms. These optimization measures can reduce the temperature and stress and verify the effectiveness of the algorithm and the feasibility of the optimization strategy [19]. In the study of heat transfer and combustion processes in piston–cylinder assemblies, numerical simulation techniques play an important role. Numerical simulation based on FEA can analyze the thermal stress and heat loss of the piston at different temperatures [20]. Especially for the analysis of static temperature field distribution and thermomechanical coupling stress, it reveals the main role of mechanical loads in piston stress and realizes the simultaneous reduction of piston mass and maximum thermomechanical coupling stress through multi-objective optimization design [21].

Although the above studies have made significant progress in multiple aspects of piston performance improvement, there are still some problems that need to be solved urgently. Most of the existing studies focus on the optimization of a single performance of the piston or the solution of a specific failure mechanism, only focusing, for example, on the improvement of wear resistance, the improvement of insulation performance, or the reduction in thermal stress; there is a lack of comprehensive optimization research on multiple performances, such as piston stiffness, strength, and dynamic characteristics. However, in actual working conditions, the various performances of the piston are interrelated and interact with each other. The improvement of a single performance may harm other performances. Hence, the multi-objective collaborative optimization is required from the system level. Meanwhile, in terms of optimization design methods, although various optimization algorithms and experimental design methods have been applied to piston design, most studies have failed to fully combine sensitivity analysis with response surface models. Sensitivity analysis can reveal the degree of influence of each design variable on objective functions, helping to clarify the optimization focus. Response surface models can efficiently establish the relationship between design variables and objective function and provide a calculation model for optimization algorithms.

This study conducts a comprehensive optimization of the stiffness, strength, and dynamic characteristics of a piston. First, a high-precision finite element model of the piston is established based on ANSYS Workbench, and the boundary conditions are determined in combination with the actual working conditions for thermomechanical coupling analysis and dynamic characteristic analysis, providing accurate data support for the optimization design. Second, five geometric dimensions are selected as design variables, and multiple performance indicators, such as deformation, mass, temperature, and the first to third natural frequencies, are taken as optimization objectives. A response surface model is constructed through the design of the experiments method, and the model is fully verified and evaluated using the “Residuals vs. Run” plot and “Predicted vs. Actual” plot. Then, a sensitivity analysis is carried out to deeply analyze the influence of each variable on the system performance in combination with the response surface. Finally, advanced optimization algorithms such as the multi-objective genetic algorithm (MOGA) are used for multi-objective collaborative optimization to improve the comprehensive performance of the piston, providing a solution for promoting the further development of engine technology.

## 2. Finite Element Analysis of the Piston

### 2.1. Establishment of the Finite Element Model of the Piston

**Figure 1:**
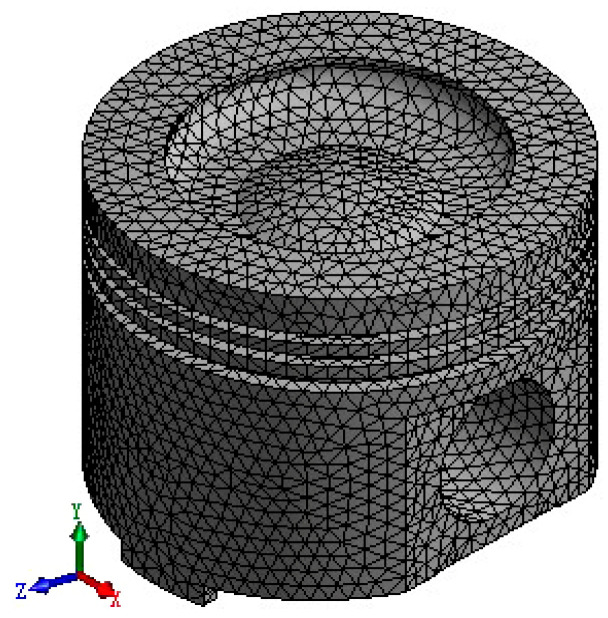
Finite element model of the piston.

This paper adopts the seamless integration of three-dimensional (3D) modeling software SolidWorks (version: 2020) and ANSYS Workbench (version: 2020 R2) to achieve efficient sharing and conversion of design data. Through this process, the established 3D piston model is directly imported into the ANSYS Workbench environment. Therefore, the integrity and accuracy of the model are preserved, and the complexity of data transmission is greatly simplified. In ANSYS Workbench, a fine meshing strategy is implemented for this model, using 5 mm as the mesh reference size to ensure the balance between analysis accuracy and computational efficiency. The divided finite element model shows highly refined structural features, including 63,689 nodes and 39,770 mesh elements, with an average mesh quality of up to 0.80. This indicator verifies the excellent quality of the meshing and provides a guarantee for the accuracy of subsequent simulation analysis. Figure 1 shows the piston model after grid division.

### 2.2. Physical Properties of Piston Materials

For the piston studied in this paper, the material selected is aluminum alloy (6061), which takes into account the needs of lightweight, high strength, and good thermal conductivity. Table 1 and Table 2 list, in detail, the thermophysical property parameters of this aluminum alloy material, including, but not limited to, key indicators such as density, elastic modulus, and thermal conductivity coefficient. The accurate input of these data lays a solid foundation for the subsequent thermal steady-state analysis, ensuring that the simulation results can truly reflect the thermal behavior characteristics of the piston in the working state.

### 2.3. Thermal Load Analysis of the Piston

In the thermal load analysis, first, we import the piston geometric model, add the corresponding material properties, and then perform mesh generation. In the mesh generation stage, an appropriate mesh strategy is adopted to refine the key areas to capture the subtle changes in heat transfer. Then, according to the actual situation, combined with the heat transfer coefficient at the surface temperature of each part of the piston, a complete thermal analysis boundary is constructed via empirical formula, as shown in Table 3 [22].

**Figure 2:**
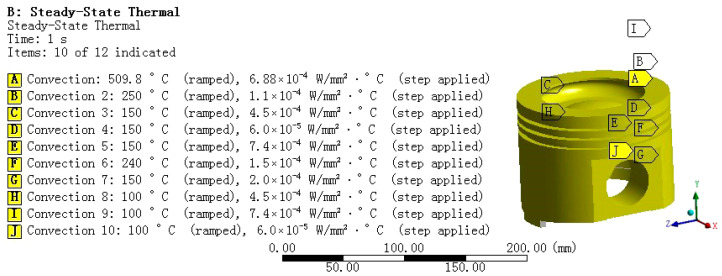
Setting of boundary conditions for the piston heat transfer.

In the specific operation, to accurately find the setting position of adding convection coefficient, we input the convection coefficient values corresponding to each part of the piston in Table 3 one by one, as shown in the Figure 2. After the convection coefficient is set, the system will perform thermal analysis and calculation on the piston according to these parameters, thus obtaining key information such as the temperature distribution of the piston in the steady thermal environment.

**Figure 3:**
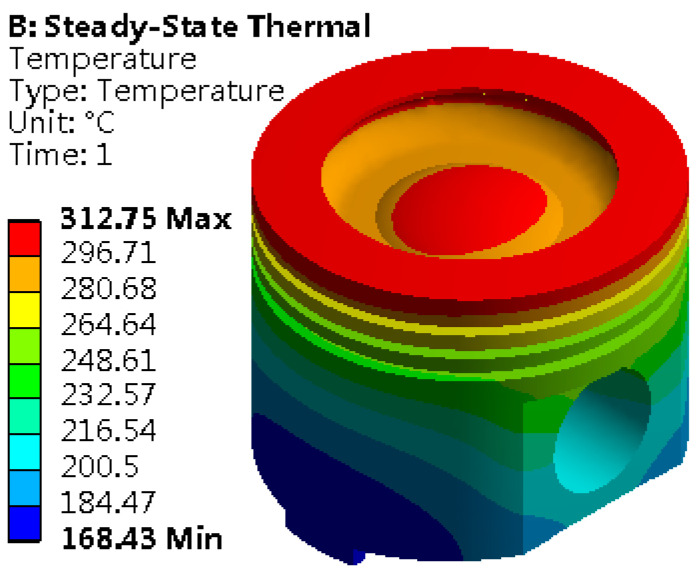
Distribution map of the piston temperature field.

Set the solution options, focusing on the core indicator of the temperature field distribution. Finally, the solution program is executed, and the temperature field distribution map is obtained, as shown in Figure 3. It reveals the temperature gradient and heat transfer path inside the piston, providing solid data support for subsequent thermal design and optimization.

The distribution map of the piston temperature field clearly reveals the spatial difference of its thermal state, with the highest temperature reaching 312.75 °C and the lowest temperature at 168.43 °C. It displays a significant temperature gradient from top to bottom. This temperature change is particularly obvious in the piston combustion chamber and the top edge area. The top edge bears the heat radiation and convection heat transfer of high-temperature gas, and the lubrication condition is limited, so it becomes the concentrated area of heat load. As the core area of gas combustion, the combustion chamber continuously receives the heat transfer of high-temperature gas, which causes the temperature in this area to climb to 312.75 °C. On the other hand, the piston skirt can effectively take away heat because they are far away from the high-temperature gas heat source, so the temperature is maintained at a low level and the skirt temperature is stable at 168.43 °C. This temperature distribution law is highly consistent with the design concept of “top heat absorption–skirt heat dissipation” of the piston. From the heat conduction path, heat is absorbed from the top of the piston and transferred to the skirt, which further accelerates the heat dissipation and forms a top-down temperature gradient. It not only verifies the accuracy of theoretical analysis but also truly restores the heat conduction and heat dissipation characteristics under actual working conditions. Meanwhile, the maximum temperature at the top, 509.8 °C, listed in Table 3, was input as a boundary condition into the simulation model, representing the external heat source temperature applied to the top of the piston during the thermal analysis process. The temperature distribution of 312.75 °C, shown in Figure 3, is the actual temperature achieved at the top of the piston after thermal steady state analysis, reflecting the true temperature situation of the model under steady-state conditions.

### 2.4. Thermal–Mechanical Coupling Analysis of the Piston

**Figure 4:**
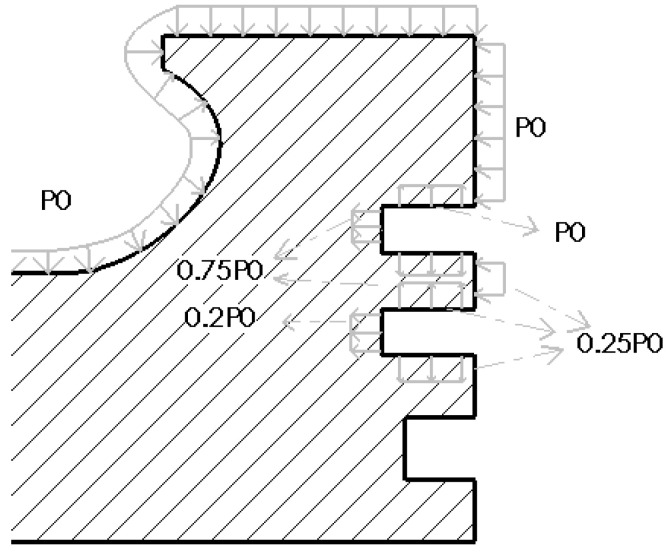
Schematic diagram of the distribution of external loads on the piston.

When the piston is subjected to gas pressure, its pressure distribution shows a significant gradient change, gradually decaying from the top to the skirt of the piston. Specifically, the pressure distribution can be finely divided into several key areas for in-depth analysis. The top surface of the piston, as the direct surface of gas pressure, bears the maximum pressure load. Next is the firepower bank area, located below the top surface, where although the pressure is reduced, it is still at a relatively high level, posing an important test of the piston strength. Then there is the three-ring area, where the pressure is further reduced, but it is still a key influencing area for the piston’s sealing and lubrication performance. Finally, the remaining parts can be regarded as negligible areas due to the relatively small pressure. Figure 4 depicts this pressure distribution characteristic in detail, providing a data basis for the mechanical analysis.

In the thermal mechanical coupling analysis of pistons, the main external loads considered are combustion gas pressure and reciprocating inertial force. Among them, the reciprocating inertial force reaches its maximum value when the piston is located at the top and bottom dead center positions. This article simulates it by applying a downward acceleration load, which is 1469 mm/s^2^.

The gas pressure load is applied according to the pressure distribution shown in Figure 4, and the specific settings are as follows.

① Surface pressure load (discretized according to the dynamic pressure curve in Figure 4).

The top surface of the piston and the upper side of the first ring groove: P_0_ = 14.7 MPa.

The inner diameter surface of the first ring groove is 0.75 P_0_ = 11.025 MPa.

The lower side of the first ring groove and the upper side of the second ring groove: 0.25 P_0_ = 3.675 MPa.

The inner diameter surface of the second ring groove is 0.2 P_0_ = 2.94 MPa.

② Inertial load

Apply a global downward acceleration of α = 1469 mm/s^2^ (simulating the maximum inertial force at the top dead center).

③ Piston pin connection constraint

Establish a cylindrical joint between the center of the piston pin hole and the fixed reference point. Designate the inner surface of the pin hole as the reference surface for moving parts (allowing rotation around the pin axis).

④ Auxiliary coordinate system

Establish a cylindrical coordinate system based on the piston top surface.

⑤ Piston skirt constraint

Apply cylindrical support to the outer surface. Fixed radial degrees of freedom (Radial DOF), releasing axial/circumferential degrees of freedom.

**Figure 5:**
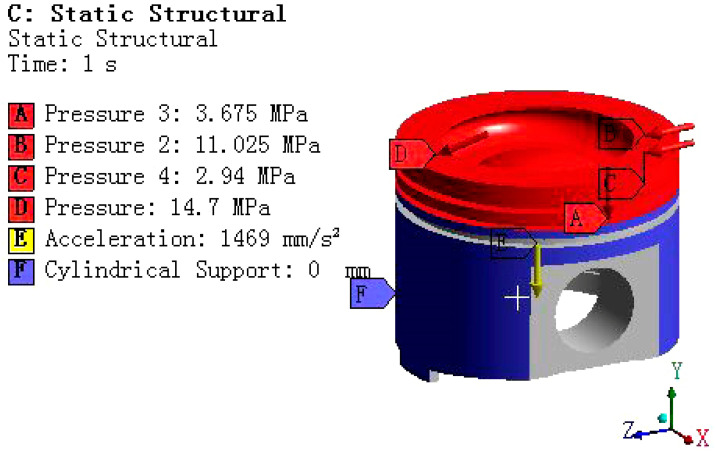
Piston mechanics boundary conditions.

The piston model with complete boundary conditions (including gas pressure, inertial load, cylindrical support, and cylindrical pair) is shown in the following Figure 5.

In addition, although this article did not directly model the interaction between the piston ring and the cylinder liner in the model, the indirect effects of the piston ring on the piston heat conduction path, contact pressure distribution, and friction behavior have been considered through the above boundary condition settings. In the subsequent structural dynamics analysis, these boundary conditions will help to more accurately reflect the thermal mechanical response of the piston under actual operating conditions.

**Figure 6:**
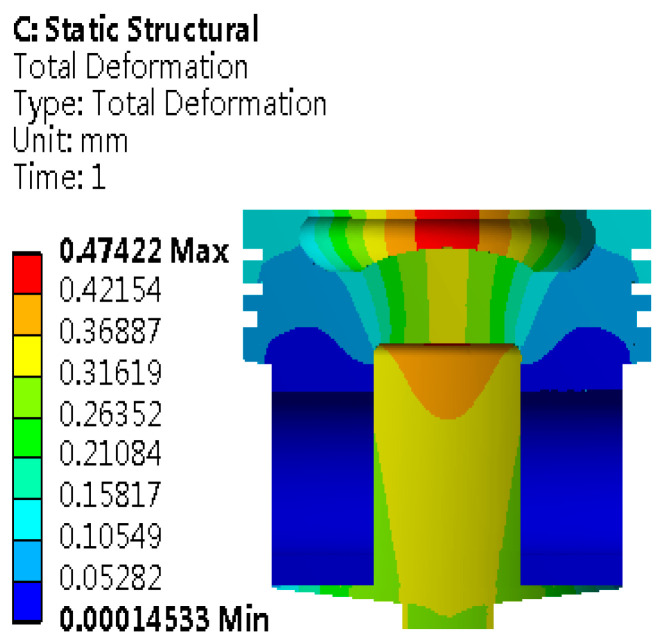
Deformation distribution map of the piston under the coupling field.

Based on theoretical analysis and adding reasonable boundary conditions. The simulation calculation is carried out. After a series of rigorous calculations and simulations, the piston deformation distribution map as shown in Figure 6 is obtained. The map visually displays the mechanical response of the piston under specific working conditions.

As shown in Figure 6, the maximum deformation reaches 0.47422 mm, and the deformation is mainly concentrated in the top area of the piston. The piston head is the core area that bears the heat load of gas. Even if the maximum deformation is considered, the piston design can still ensure the effective sealing performance between the cylinder liner and the piston. Therefore, the piston is always in a stable working state, and cylinder pulling failure caused by its deformation is effectively avoided. In the actual working environment, this design ensures the dynamic balance between the piston and the cylinder liner, preventing friction and wear caused by too small a gap and avoiding the problem of reduced sealing performance due to too large a gap, laying the foundation for the continuous and efficient operation of the piston. Therefore, the piston can effectively resist the deformation effect caused by the thermal load, ensuring that the engine is not disturbed by this factor and maintaining its normal operating state.

### 2.5. Modal Analysis of the Piston

**Figure 7:**
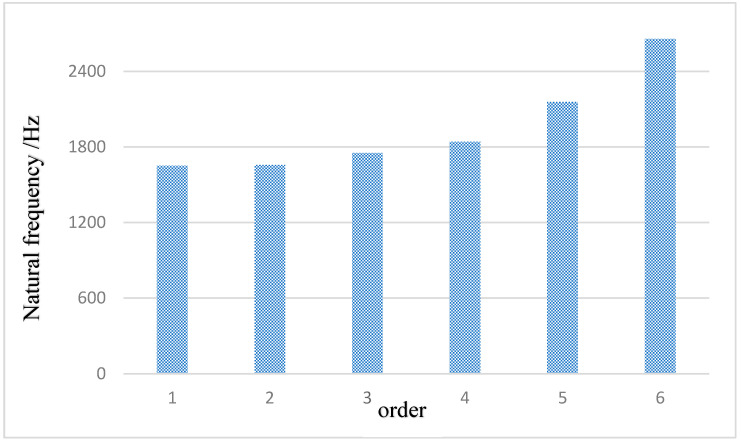
Modal analysis results.

Modal analysis, as a key means to study the dynamic characteristics of structures in the state of free vibration, aims to research the natural frequencies and modal shapes. In the structural design of pistons, this analysis link is particularly important as it directly relates to the vibration performance and operational stability of the piston. By using ANSYS software (version: 2020 R2), the first- to sixth-order natural frequencies were obtained, as shown in Figure 7. It displays the vibration patterns of the piston at different orders, providing data support for the in-depth understanding of the dynamic response characteristics of the piston, optimizing structural design, and preventing resonance phenomena. It is an indispensable part of piston performance evaluation and optimization design.

The first- to sixth-order natural frequencies of the piston are shown in Table 4 below.

This study conducts a detailed modal analysis of the piston, aiming to accurately solve its natural frequencies and corresponding vibration modes at each order. This analysis not only provides a scientific basis for the dynamic performance evaluation of the piston but also prevents fatigue fracture problems caused by resonance during the piston’s working process by identifying potential resonance risks. Therefore, it focuses on the in-depth analysis of the first to sixth orders of vibration modes. The natural frequency distribution of the piston presents certain characteristics, in which the high-order frequencies are significantly higher than 1651.6 Hz, while the low-order natural frequencies are densely distributed in a narrow range from 1651.6 Hz to 2656.8 Hz. Based on the conclusion of the modal analysis, it is of great significance for the subsequent optimization design of the piston structure, aiming to reduce the possibility of resonance and ensure the long-term stable operation.

### 2.6. Harmonic Response Analysis of the Piston

Harmonic response analysis, also known as sweep frequency analysis, is a key means to evaluate the steady-state response of linear structures under periodic external forces. This analysis focuses on the forced vibration characteristics of the structure, aiming to accurately define its vibration frequency range and response mode. Through this method, it is possible to effectively capture and reflect the resonance phenomenon and potential fatigue risks of the structure at specific frequencies, providing a scientific basis for optimizing design and safe operation. In practical operations, the modal superposition method, as the core technology of harmonic response analysis, is widely used in the calculation process. In this method, the modal shapes of each order of the structure are weighted and superimposed according to the corresponding coefficients. This method is used to evaluate the overall dynamic response of the structure under different frequency excitations so as to improve the accuracy of analysis and make the calculation results closer to the actual situation. It also significantly enhances the understandability and interpretability of the results.

**Figure 8:**
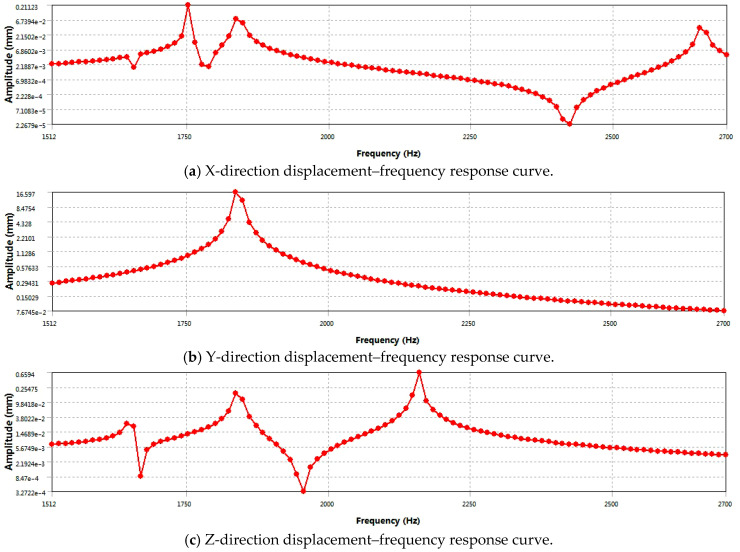
Displacement–frequency response curve of the piston.

To deeply explore the dynamic characteristics of the piston, this study takes modal analysis as the theoretical basis and adopts the modal superposition method to analyze the harmonic response of the piston. To simulate the actual working condition, the piston pin is fixed, and the first to sixth modes are extracted via the Block Lanczos method, focusing on the modal characteristics in the frequency band of 1500–2700 Hz. A pressure load of 14.7 MPa in the -Y direction was applied to the piston top surface, the maximum scanning frequency was set at 2700 Hz, and a fine frequency interval of 100 Hz was adopted. Based on the first to sixth natural frequencies obtained by modal analysis, the displacement–frequency response curves in the X, Y, and Z directions were drawn, as shown in Figure 8. These curves demonstrate the displacement response law of the piston under different frequency excitations, providing important data support for predicting the resonance behavior, evaluating the structural strength, and optimizing the design scheme.

The results of harmonic response analysis provide key data support for the dynamic performance analysis of the piston. Figure 8a reveals the displacement behavior of the piston in the X direction, showing that at a specific frequency of 1752 Hz, the displacement response reaches a peak and then gradually decays, indicating that the vibration of the piston is the most intense at this frequency. Figure 8b shows that there is a peak displacement of 16.6 mm near the resonance frequency of 1840 Hz, which represents the maximum relative displacement component achieved by the piston at this excitation frequency. This displacement reflects the vibration amplitude of the piston in the Y-axis direction caused by excitation and is an important indicator of structural dynamic response. Figure 8c shows the vibration characteristics of the piston in the Z direction. At the frequency of 2160 Hz, the vibration displacement reaches its maximum and then decreases gradually. It shows that the resonance frequency in the Z direction is different from the first two.

Based on the results of harmonic response analysis, the piston geometry studied in this paper presents remarkable vibration characteristics under dynamic load. In the spatial rectangular coordinate system, three characteristic resonance frequencies of 1752 Hz (X direction), 1836 Hz (Y direction), and 2160 Hz (Z direction) are detected, respectively. Therefore, based on the above analysis of dynamic response characteristics, although the resonance frequency characteristics (1752 Hz/1836 Hz/2160 Hz) revealed in this study are only applicable to the specific piston geometry studied in this paper, the harmonic response analysis method based on modal analysis results can provide a universal technical path for the evaluation of vibration characteristics of similar reciprocating components. It is suggested that the above characteristic frequency range should be strictly avoided in the design of engine operating conditions and the control of the piston excitation source in order to prevent the risk of fatigue failure caused by co-vibration stress. For piston components with similar topological structure characteristics or service conditions, the whole process analysis framework of “geometric modeling-modal analysis-frequency response prediction” constructed in this study can be used for reference to carry out targeted structural dynamics optimization design.

## 3. Optimization Design of the Piston

### 3.1. Mathematical Model and ANOVA

The quadratic response surface optimization mathematical model is an optimization method based on the multivariate quadratic regression equation in statistics to fit the complex functional relationship between design variables and objective functions. It achieves the purpose of reducing the amount of calculation and improving the optimization efficiency by constructing a simplified quadratic polynomial model to replace the highly complex input-output relationship in the actual system. The quadratic response surface model is based on the multiple linear regression method and determines the unknown coefficients in the polynomial by the least squares estimation method. This model represents the system’s response (i.e., output variable or objective function) as a quadratic polynomial function of the design variables (i.e., input parameters).

When optimizing the design of the piston, selecting and defining the optimization variables is a crucial first step. To achieve the optimization of piston performance, five geometry dimensions were selected as design variables: *x*_1_ is the height of the fire land; *x*_2_ is the top surface width; *x*_3_ is the top surface thickness; *x*_4_ is radius of top surface transition arc 2; and *x*_5_ is radius of top surface transition arc 1. The selection of these parameters is based on their significant impact on the working performance of the piston, such as sealing, heat load capacity, mechanical strength, and durability. Table 5 details the initial value ranges of these design variables.

**Figure 9:**
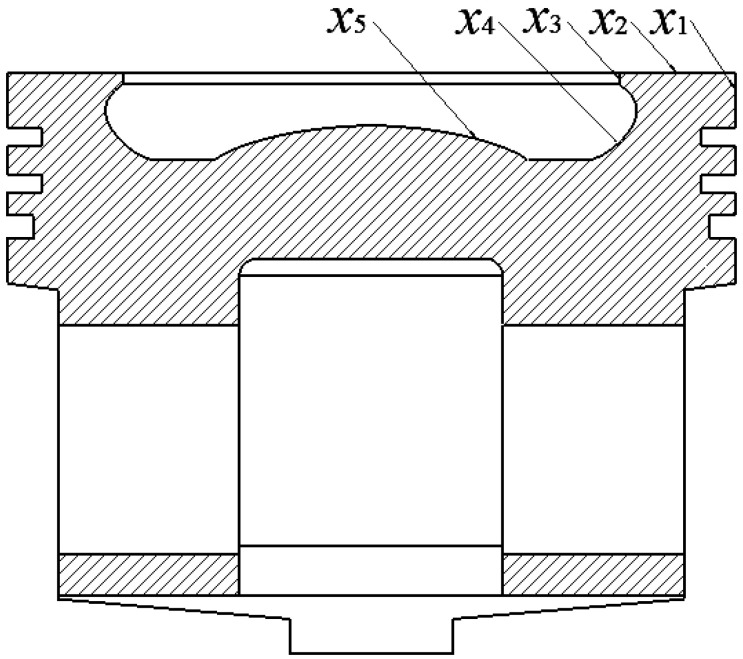
Design variables.

Figure 9 shows the geometric structure of the piston and marks the specific locations of these geometric dimensions. By adjusting the values of these design variables, this study aims to explore the potential for improving piston performance without increasing additional material costs or manufacturing complexity.

The response surface analysis was conducted on the designed sample points by using Design-Expert software (version: 8.0.6). First, the sample points were accurately input into the system, and then the software automatically processed and generated detailed calculation expressions. This process not only simplifies the complex data processing flow but also ensures the accuracy of the analysis results. Subsequently, through the built-in variance analysis function, the software comprehensively evaluated the significance of the regression model equation, and the results showed that the model fitting effect was excellent, providing a solid statistical basis for subsequent in-depth analysis. It is particularly worth noting that Design-Expert visually displays the regression equation with encoded independent variables as the core, which profoundly reveals the intrinsic relationship between design variables and performance goals.

For the quadratic polynomial response surface model of n design variables, as shown in Equation (1).

(1) $$y=\beta_{0}+\sum_{i=1}^{n}\beta_{i}x_{i}+\sum_{i=2}^{n}\sum_{j=1}^{i-1}\beta_{ij}x_{i}x_{j}+\sum_{i=1}^{n}\beta_{ii}x_{i}^{2}$$

In the formula, *y* is the objective function.

*β*_0_, *β_i_*, *β_ij_*, and *β_ii_* (*i* < *j*) are undetermined coefficients of the polynomial.

*β*_0_ is a constant term.

*β_i_* is the linear effect of the design variable *x_i_*.

*β_ij_* is the linear interaction effect between design variables *x_i_* and *x_j_*.

*β_ii_* is the quadratic effect of the design variable *x_i_*.

*n* is the number of design variables.

Generally, the quadratic response surface modeling has a 95% possibility. Due to the large number of design variables of the moving crossbeam, the quadratic response surface is directly used for modeling, and all quadratic terms, linear terms, and interactions between various factors are considered. In the quadratic regression analysis, the backward method (Backward) is used to delete the design variables and interactions that have no significant impact. Through the above analysis, the final established quadratic response surface models are shown in Equations (2)–(7).

The final equation of mass is fitted by the quadratic response surface model, as shown in *y*_1_.

(2) $$\begin{array}{l} y_{1}=0.37+0.06x_{2}-3.14\times 10^{-3}x_{3}-9.48\times 10^{-3}x_{4}-0.01x_{5}+1.07\times 10^{-3}x_{2}x_{3}+\\ \;1.36\times 10^{-4}x_{2}x_{5}-1.92\times 10^{-4}x_{4}x_{5}+3.62\times 10^{-4}x_{2}^{2}+2.37\times 10^{-4}x_{4}^{2}+1.24\times 10^{-4}x_{5}^{2} \end{array}$$

The final equation of the temperature fitted by the quadratic response surface model, as shown in *y*_2_.

(3) $$\begin{array}{l} y_{2}=383.8-3.07x_{1}-10.41x_{2}-5.59x_{3}+0.42x_{4}+3.22x_{5}-\\ \;0.1x_{2}x_{5}+1.66x_{1}^{2}+0.29x_{2}^{2}+1.37x_{3}^{2}-0.01x_{4}^{2}+0.04x_{5}^{2} \end{array}$$

The final equation of the deformation is fitted by the quadratic response surface model, as shown in *y*_3_.

(4) $$y_{3}=1.57-0.1x_{1}-0.06x_{2}-0.01x_{3}+6.97\times 10^{-4}x_{4}-4.57\times 10^{-4}x_{5}+5.49\times 10^{-3}x_{1}^{2}+1.28\times 10^{-3}x_{2}^{2}$$

The final equation of the first frequency is fitted by the quadratic response surface model, as shown in *y*_4_.

(5) $$y_{4}=-5526.51+668.01x_{2}+7.31x_{3}+2.68x_{4}-17.26x_{5}-14.87x_{2}^{2}$$

The final equation of the second frequency is fitted by the quadratic response surface model, as shown in *y*_5_.

(6) $$y_{5}=-7679.99+1565.04x_{2}+2.33x_{4}-672.41x_{5}+34.66x_{2}x_{5}-56.02x_{2}^{2}$$

The final equation of the third frequency is fitted by the quadratic response surface model, as shown in *y*_6_.

(7) $$y_{6}=868.72-438.46x_{2}+486.76x_{5}-26.05x_{2}x_{5}+25.71x_{2}^{2}$$

To evaluate the fitting ability of the quadratic response surface model to the output response, the F-value, P-value, Std. Dev (standard deviation), Mean (mean), Mean Square (mean square), Sum of Squares and Residual of the model are given, respectively. At the same time, the determination coefficient *R*^2^, the adjusted determination coefficient *R_a_*^2^, and the prediction determination coefficient *R_pred_*^2^ are used for evaluation. The calculation methods of the determination coefficient *R*^2^ and the adjusted determination coefficient *R_a_*^2^ are given in Equations (8) and (9).

(8) $$R^{2}=1-\frac{S_{\mathrm{SE}}}{S_{\mathrm{ST}}}$$

(9) $$R_{a}^{2}=\frac{\frac{S_{\mathrm{SE}}}{P-L-1}}{\frac{S_{ST}}{P-1}}$$

In the equations,

(10) $$S_{\mathrm{SE}}=\sum_{i=1}^{P}{\left(Y_{i}-y_{i}\right)}^{2}$$

(11) $$S_{\mathrm{ST}}=\sum_{i=1}^{P}Y_{i}^{2}-\frac{{\left(\sum_{i=1}^{P}y_{i}\right)}^{2}}{P}$$

The evaluation value table of the fitting degree of the response surface model is shown in Table 6.

**Figure 10:**
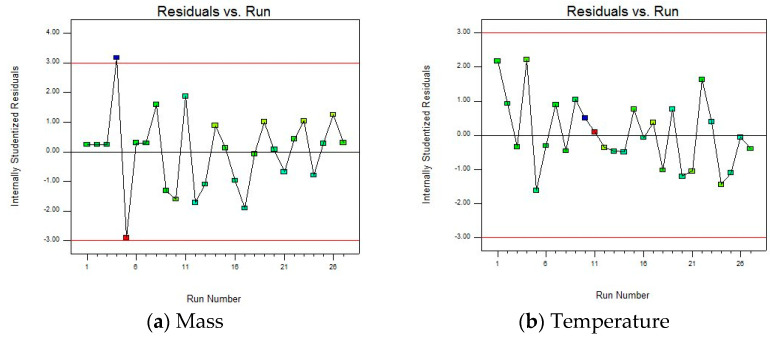

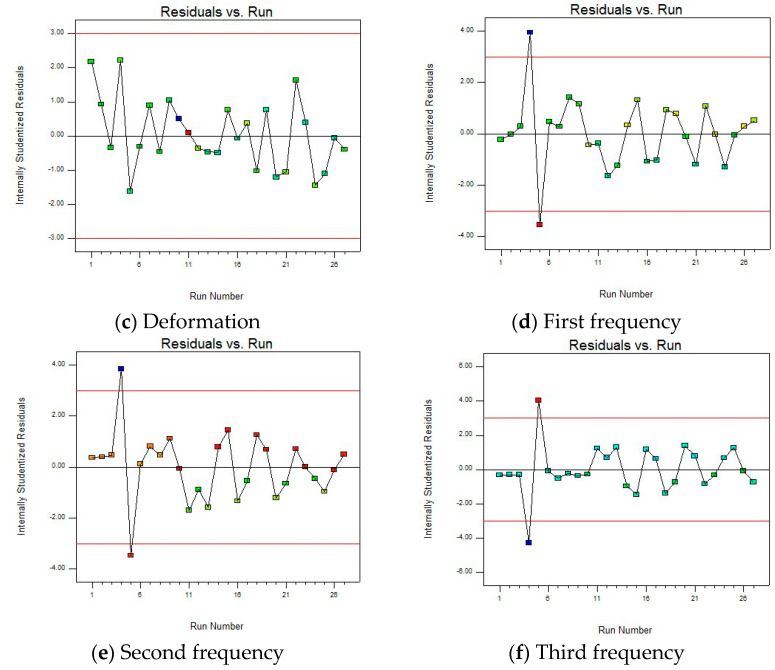
Residuals vs. run of the model.

To study the rationality of the fitted model, the sufficiency of model fitting, and to identify potential data problems, the “Residuals vs. Run” plot is given, as shown in Figure 10.

There are no obvious trends, periodicities, etc., in the residual and run sequence number plot, and there are no upward, downward, or periodic change trends with the increase in the run sequence number, indicating that the model fitting effect is good and can better capture the patterns in the data. However, there are also two abnormal data points in the figure that are far from the zero value line (generally considered that the absolute value of the internal studentized residual is greater than 3, as shown by the red line in the figure), namely, 4 and 5 in the run sequence. Therefore, when optimizing the model in the subsequent steps, the outliers should be corrected or removed to improve the accuracy and reliability.

**Figure 11:**
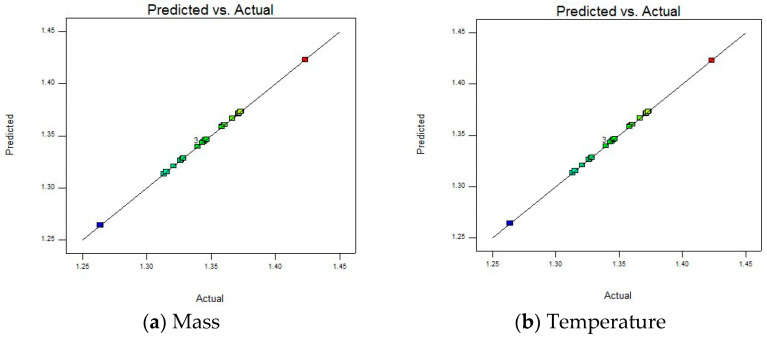

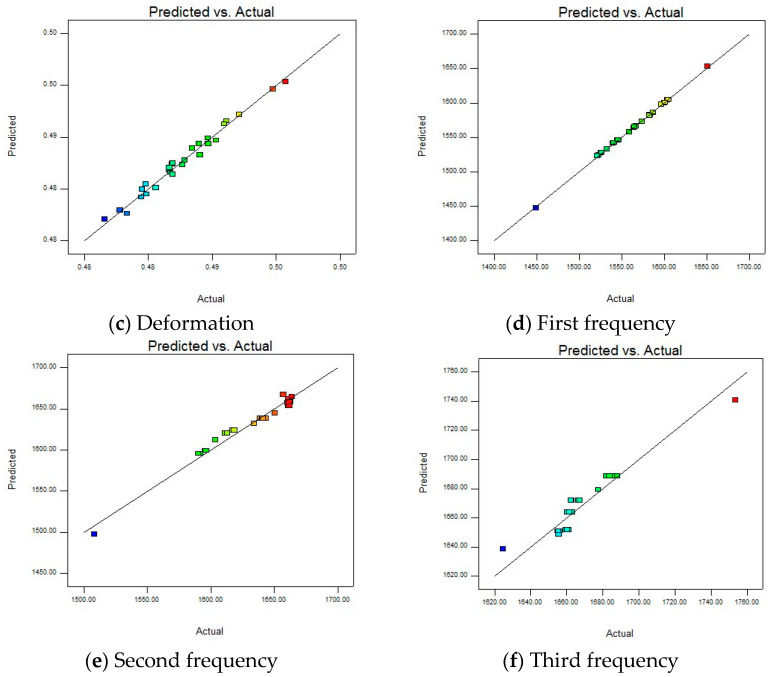
Predicted vs. actual of the model.

To further evaluate the fitting accuracy and predictive ability of the model on the experimental data, a “Predicted vs. Actual” diagram is presented, as shown in Figure 11. Studying whether the data points are distributed around the diagonal, it is apparent that the model can accurately capture the experimental patterns. This visual comparison can quickly identify whether the model is over-fitting or under-fitting, providing direction for subsequent optimization.

Most of the data points are relatively concentrated near the straight line, indicating that the predicted values of the model and the actual values are relatively close, and the model can fit the actual situation to a certain extent, which is a relatively ideal state. At the same time, there are individual points (such as blue and red points) that deviate far from the straight line. Combined with the residual and run sequence number diagram, outliers need to be corrected or eliminated to improve the accuracy and reliability of the model.

### 3.2. Sensitivity Analysis

**Figure 12:**
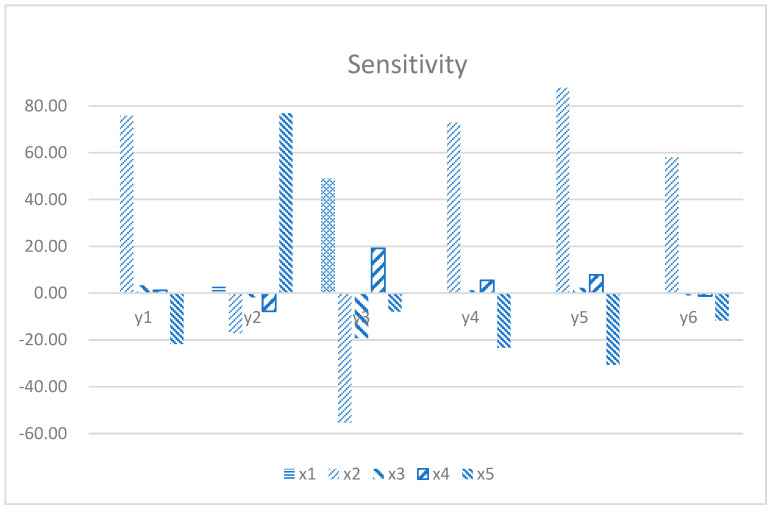
Results of sensitivity analysis.

Sensitivity analysis, as a core analytical method, aims to precisely quantify the sensitivity of the output of a mathematical model or system to changes in its input parameters. This method delves into how the system state or output changes in response to fine-tuning system parameters and external conditions, thereby revealing which parameters have the most significant impact on system performance. Based on the results of a detailed design of experiments (DOE), by analyzing the generated design points, this study successfully plotted the sensitivity histogram of the five geometry dimensions as shown in Figure 12. It directly shows the contribution of each parameter to the model output change and provides valuable insight for decision-makers and researchers, enabling them to focus on those parameters that have the greatest impact on the model performance. Based on these sensitivity analysis results, targeted model optimization strategies can be implemented to significantly improve the prediction accuracy of the model.

In the analysis results presented in Figure 11, the significant impact of multiple key parameters of piston design on its performance indicators can be clearly observed. *x*_2_ and *x*_5_ are crucial in optimizing piston performance. First, these two parameters have a significant impact on *y*_1_, indicating that in lightweight design, precisely regulating the top surface width and the size of the transition arc is an effective way to reduce piston mass. *x*_2_ and *x*_5_ have the same important impact on *y*_2_. By optimizing these parameters, the heat load distribution of the piston during operation can be effectively adjusted, thereby reducing the piston temperature, extending the service life of the material, and improving the overall thermal efficiency of the engine. The mechanical response characteristics of the piston are also jointly regulated by multiple design parameters. Specifically, *x*_1_, *x*_2_, *x*_3_, and *x*_4_ jointly act on *y*_3_. In terms of dynamic characteristics, the changes in *x*_2_ and *x*_5_ show significant effects on *y*_4_, *y*_5_, and *y*_6_. The increase in natural frequency helps to enhance the vibration resistance of the piston, reduce the risk of failure caused by resonance, and ensure the smoothness and reliability of engine operation. Through reasonable combination and collocation of these parameters, the deformation of piston can be reduced. In this way, the structural strength of the piston is effectively enhanced, and the risk of fatigue failure caused by stress concentration phenomenon is significantly reduced. Thereby effectively improving its durability and reliability. The sensitivity analysis results reveal the internal relationship between piston design parameters and key performance indexes. At the same time, it points out the direction for the subsequent optimization design. By reasonably changing the parameters *x*_1_ to *x*_5_, the piston temperature can be reduced, and the dynamic characteristics can be optimized.

### 3.3. Results and Discussion

Based on sensitivity analysis results, the response surface methodology is used to construct a complex mathematical model between the objective function (such as mass *y*_1_, temperature *y*_2_, and maximum deformation *y*_3_, etc.) and the design variables (*x*_1_, *x*_3_, *x*_3_, *x*_4_, and *x*_5_). This model aims to accurately describe the nonlinear relationship between parameters and provide strong theoretical support for optimization design.

Since the response surface model used in this paper involves five design variables and six objective functions, it will take a lot of space to list all model expressions completely, and not all models have significant engineering analysis value. Therefore, the response surface models selected are all developed around the scenes where there is a significant correlation between the design variables and the objective functions. In the meantime, combined with the results of sensitivity analysis, the variable combinations that show strong parameter sensitivity in mathematics are screened out together, and the shape of response surface is presented intuitively.

**Figure 13:**
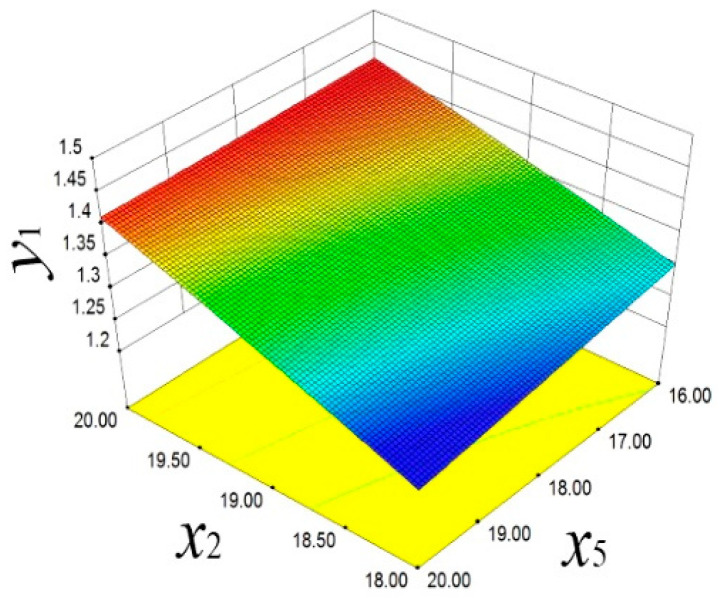
Mass response surface.

**Figure 14:**
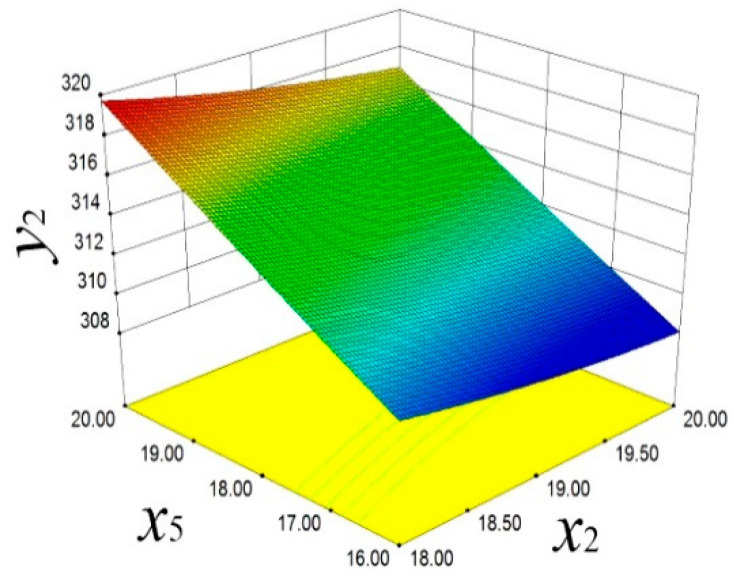
Temperature response surface.

As shown in Figure 13, the response surface model reveals a positive correlation between *x*_2_ and *y*_1_. With the increase in *x*_2_, *y*_1_ significantly increases. The decrease in *x*_5_ also has a similar effect on *y*_1_, indicating that these two parameters have significant sensitivity in controlling the piston mass. As shown in Figure 14, the reverse regulation effect of *x*_2_ and *x*_5_ on the piston temperature *y*_2_ is demonstrated. When *x*_2_ decreases and *x*_5_ increases, *y*_2_ shows an upward trend. This finding reveals the important role of design variables in regulating the distribution of piston thermal load, providing a scientific basis for optimizing the thermal management strategy.

**Figure 15:**
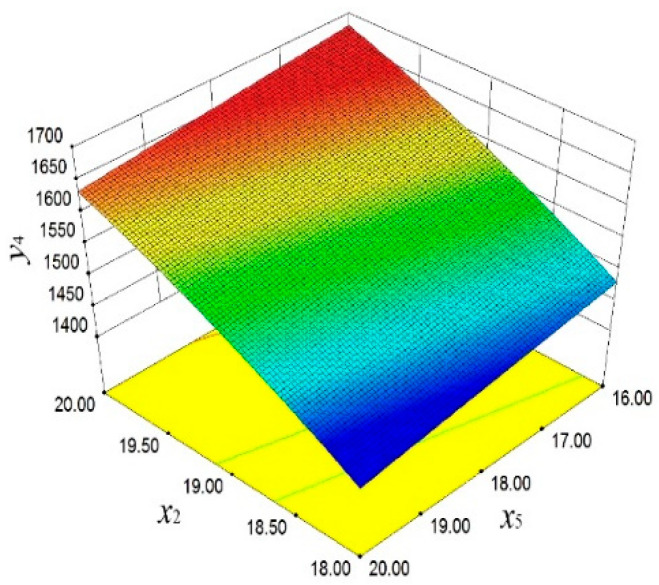
First-order frequency response surface.

**Figure 16:**
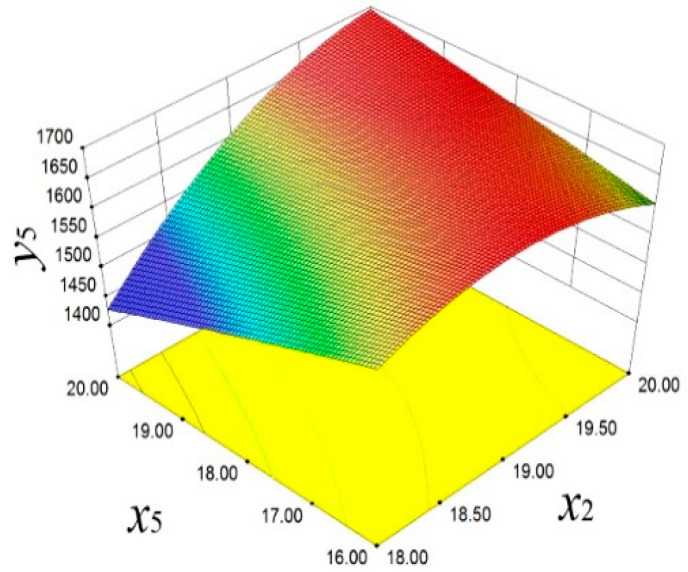
Second-order frequency response surface.

In Figure 15, the significant influence of *x*_2_ and *x*_5_ on *y*_4_ is demonstrated. With the increase in *x*_2_ and the decrease in *x*_5_, *y*_4_ shows an upward trend. This finding emphasizes the importance of these two design parameters in improving the dynamic stability of the piston. Figure 16 reveals the positive effect of *x*_2_ and *x*_5_ on *y*_5_. With the increase in *x*_2_ and the decrease in *x*_5_, *y*_5_ also increases.

**Figure 17:**
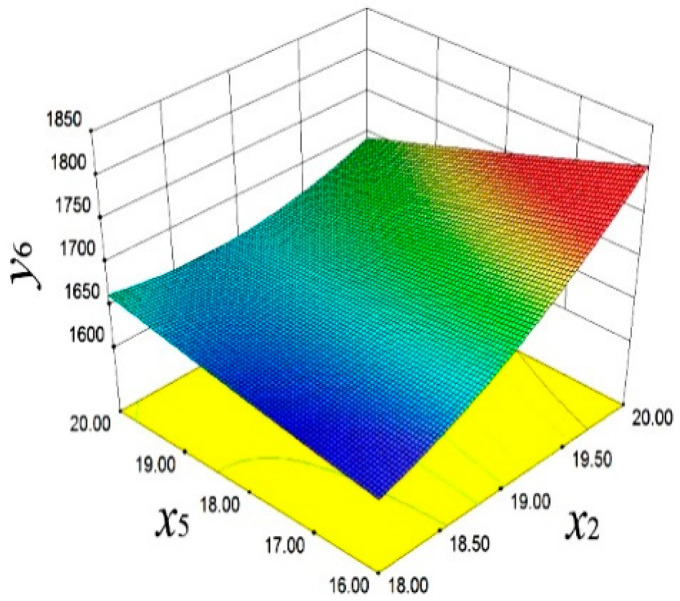
Third-order frequency response surface.

**Figure 18:**
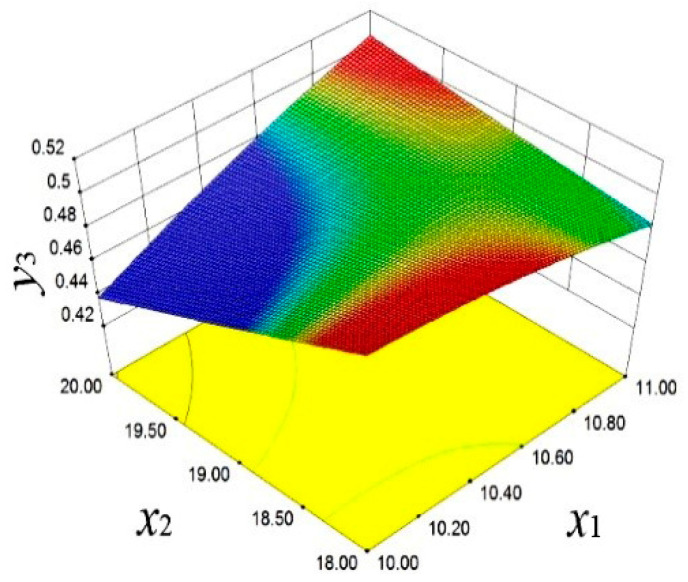
Deformation response surface.

In Figure 17, the influence of *x*_2_ and *x*_5_ on *y*_6_ is demonstrated. Specifically, with the increase in *x*_2_ and *x*_5_, *y*_6_ significantly increases. This finding reveals the crucial role of design variables in adjusting the high-order vibration characteristics, providing a perspective for improving the smooth operation of the engine. As shown in Figure 18, *y*_3_ is affected by x_2_ and *x*_1_. When *x*_2_ and *x*_1_ decrease, *y*_3_ significantly increases.

This complex relationship indicates that in optimizing the piston structure to reduce deformation and improve stiffness, the synergistic effect of multiple design parameters needs to be considered to achieve the best design effect.

The results explores the balance between mass, temperature, deformation, and the first to third frequencies, achieving a significant improvement in its overall performance without increasing mass. Then, the MOGA, screening, and RSM were, respectively, used to conduct a comprehensive and in-depth optimization. Through numerical calculations, the results of different optimization algorithms are given, as shown in Table 7.

After elaborate experiments and in-depth analysis, the MOGA variation algorithm was finally selected as the optimal solution for implementation. The optimized results show that on the premise of relatively stable mass and deformation, a significant reduction in temperature has been achieved. The working temperature of the piston has been effectively reduced from the initial 312.75 °C to 308.07 °C. This improvement not only extends the service life of the component but also enhances the thermal efficiency of the overall system. In terms of dynamic characteristics, the optimized piston shows better dynamic characteristics. The first, second, and third frequencies are increased from 1651.60 Hz, 1656.70 Hz, and 1665.70 Hz to 1671.80 Hz, 1665.70 Hz, and 1776.50 Hz, respectively. The first to third frequencies are improved. The dynamic stability and vibration resistance of the piston are further enhanced.

To sum up, the optimization method proposed in this paper reduces the working temperature, improves its natural frequency and overall dynamic performance, and achieves the expected goal of multi-objective optimization design. The research results provide a method for the optimal design of the piston and even internal combustion engine parts.

## 4. Conclusions

This research focuses on the performance improvement and reliability enhancement of the piston, and through a series of systematic analysis and optimization processes, significant research results have been achieved. In the model construction and analysis stage, this study fully utilized the powerful functions of SolidWorks 3D modeling software to achieve the rapid construction and adjustment of the piston parametric model, providing a strong guarantee for the flexibility and efficiency of optimization design. At the same time, with the help of the precise calculation ability of ANSYS FEA software, this study deeply explored the temperature and deformation distribution under working conditions, visually displayed its distribution map, providing an intuitive and scientific basis for the evaluation and optimization of structural strength. In addition, the application of modal analysis and harmonic analysis further revealed the inherent vibration characteristics of the piston, providing an important reference for preventing resonance and ensuring operational stability. In terms of optimization strategies and methods, this study combines DOE experimental design and response surface optimization methods. Through scientific experimental design and implementation, a large amount of data has been collected and analyzed, and a highly accurate regression equation model has been constructed. This model not only accurately captures the complex relationship between design variables and objective function but also provides a clear path for the formulation of optimization schemes through response surface analysis. On the premise of maintaining relatively stable mass and deformation, the optimized piston achieves a significant reduction in working temperature. At the same time, the optimization of dynamic characteristics further enhances its operational stability and vibration resistance.

## Figures and Tables

**Table 1:** Material attribute values.

Item	Density	Elastic Modulus	Poisson’s Ratio	Tensile Strength	Compressive Strength
Value	2770 kg/m^3^	71 GPa	0.33	268 MPa	260 MPa

**Table 2:** Thermal expansion coefficient and thermal conductivity coefficient.

Temperature/°C	Thermal Conductivity Coefficient (W·m^−1^·°C^−1^)	Thermal Expansion Coefficient (°C^−1^)
20	145.9	19.2 × 10^−6^
100	153.8	19.2 × 10^−6^
150	156.9	19.2 × 10^−6^
200	158.5	20.5 × 10^−6^
250	159.2	20.5 × 10^−6^
300	159.4	21.23 × 10^−6^

**Table 3:** Thermal boundary conditions of piston.

Location	Temperature/°C	Heat Transfer Coefficient/W·(m^2^·°C)^−1^
Top	509.8	688
Firepower shore	250	110
On the first ring groove	150	450
In the first ring groove	150	60
Under the first ring groove	150	740
First ring bank	240	150
Second ring bank	150	200
On the second and third ring groove	100	450
Under the second and third ring groove	100	60
In the second and third ring groove	100	740
Outer side of skirt	90	300
Inner side of skirt	85	90

**Table 4:** First to sixth order natural frequency of the piston.

Order	Frequency/Hz	Mode Description
1	1651.6	The whole piston swings left and right along the X-axis, and the piston skirt is deformed greatly.
2	1656.7	The vibration of the piston is similar to that of the first order, swinging left and right along the X-axis, and the deformation is mainly concentrated in the piston skirt.
3	1752.9	The overall deformation is large and twisted in the YZ plane, and the deformation is mainly concentrated in the piston skirt.
4	1840.9	The overall deformation of the piston is large, twisting in the YZ plane, and the deformation of the piston skirt is large.
5	2157.7	The piston is twisting in the XY plane and swinging from side to side, and the deformation is mainly concentrated in the piston skirt.
6	2656.8	The piston is bent and deformed in the XY plane and swings left and right, and the piston skirt is deformed slightly.

**Table 5:** Design variables and variation range.

	Name	Original Value	Variation Range
*x*_1_	Height of the fire land	20 mm	10 ≤ x_1_ ≤ 11
*x*_2_	Top surface width	10 mm	18 ≤ x_2_ ≤ 20
*x*_3_	Top surface thickness	2 mm	1.8 ≤ x_3_ ≤ 2.2
*x*_4_	Radius of top surface transition arc 2	27.96 mm	25.168 ≤ x_4_ ≤ 30.76
*x*_5_	Radius of top surface transition arc 1	18.71 mm	16.84 ≤ x_5_ ≤ 20.58
*y*_1_	Mass	1.42 kg	Find the minimum value
*y*_2_	Temperature	312.75 °C	Find the minimum value
*y*_3_	Deformation	0.47 mm	Find the minimum value
*y*_4_	1st frequency	1651.60 Hz	Find the maximum value
*y*_5_	2nd frequency	1656.70 Hz	Find the maximum value
*y*_6_	3rd frequency	1752.90 Hz	Find the maximum value

**Table 6:** Evaluation table of goodness of fit of response surface model.

	Mass	Temperature	Deformation	First Frequency	Second Frequency	Third Frequency
Model degree	Quadratic	Quadratic	Quadratic	Quadratic	Quadratic	Quadratic
F-value	3.24 × 10^6^	16,674.19	156.89	6156.77	164.94	52.10
P-value	<0.0001	<0.0001	<0.0001	<0.0001	<0.0001	<0.0001
	significant	significant	significant	significant	significant	significant
Std. Dev	2.64 × 10^−5^	0.02	6.93 × 10^−4^	1.12	6.21	7.17
Mean	1.34	314.15	0.49	1563.45	1631.49	1666.92
Mean Square	2.26 × 10^−3^	9.55	4.23 × 10^−5^	7775.94	6353.05	2675.17
R-squared	100%	99.99%	98.30%	99.93%	97.52%	90.45%
Adj R-squared	100%	99.99%	97.67%	99.92%	96.93%	88.72%
Pred R-squared	100%	99.96%	93.90%	99.48%	81.60%	17.78%
Adeq Precision	9445.69	687.14	46.83	132.10	57.98	33.09
Sum of Squares	0.023	105.10	2.97 × 10^−4^	38,879.69	31,765.27	10,700.69
Residual	1.17 × 10^−8^	8.60 × 10^−3^	1.01 × 10^−5^	26.52	808.87	1129.56

**Table 7:** Comparison of different optimization algorithms.

Parameters	Original Value	MOGA	Screening	RSM
*x*_1_/mm	10.00	10.02	10.23	10.00
*x*_2_/mm	20.00	19.90	19.80	19.26
*x*_3_/mm	2.00	2.00	2.10	2.01
*x*_4_/mm	27.96	25.62	27.00	27.46
*x*_5_/mm	18.71	16.87	16.87	16.96
Mass/kg	1.42	1.43	1.41	1.39
Temperature/°C	312.75	308.07	309.01	308.77
Deformation/mm	0.47	0.48	0.48	0.48
First frequency/Hz	1651.60	1671.80	1653.80	1619.95
Second frequency/Hz	1656.70	1665.70	1669.60	1663.95
Third frequency/Hz	1752.90	1776.50	1736.20	1705.01

## Data Availability

The original contributions presented in this study are included in the article. Further inquiries can be directed to the corresponding author.
